# ESBMC: Scalable and Precise Test Generation based on the Floating-Point Theory (Competition Contribution)

**DOI:** 10.1007/978-3-030-45234-6_27

**Published:** 2020-03-13

**Authors:** Mikhail R. Gadelha, Rafael Menezes, Felipe R. Monteiro, Lucas C. Cordeiro, Denis Nicole

**Affiliations:** 8 University of Paderborn, Paderborn, Germany; 9 ICREA, Open University of Catalonia, Barcelona, Spain; 10 SIDIA Instituto de Ciência e Tecnologia, Manaus, Brazil; 11 Federal University of Amazonas, Manaus, Brazil; 12 Kilburn Building University of Manchester, Oxford Rd, Manchester, M13 9PL UK; 13 University of Southampton, Southampton, UK

**Keywords:** Automated Test Generation, Bounded Model Checking, Software Testing, Satisfiability Modulo Theories

## Abstract

ESBMC is an SMT-based bounded model checker for real-world C programs. Such programs often represent real numbers using the floating-points, most commonly, the IEEE floating-point standard (IEEE 754-2008). Thus, ESBMC now includes a new floating-point arithmetic encoding layer in our SMT backend, that encodes floating-point operations into bit-vector operations. In particular, ESBMC can use off-the-shelf SMT solvers that offer support for bit-vectors only to encode floating-point arithmetic.

## 1 Test Generation Approach

ESBMC [3,7] is an SMT-based bounded model checker for the verification of safety properties and assertions in both sequential and multi-threaded C programs. ESBMC primarily aims to help software developers by finding subtle bugs in their code (e.g., array bounds violation, null-pointer dereference, arithmetic overflow, and deadlock). It also implements $k$-induction [5,10] and can be used to prove the absence of property violations, i.e., program correctness. In Test-Comp'20 [1], ESBMC produces test cases using the falsification mode, which is an iterative bounded model checking (BMC) approach that repeatedly unwinds the program until it either finds a property violation or exhausts time or memory limits. Intuitively, ESBMC aims to find a counterexample with up to $k$ loop unwindings. The algorithm relies on the symbolic execution engine to increasingly unwind the loop after each iteration. ESBMC uses the falsification mode because it is known that there exist property violations in all programs in the Test-Comp, so there exists no need to prove correctness. From the counterexample produced by ESBMC, we define the test specification required by the competition using an external Python script.

⋆ Jury member

ESBMC runs with an improved SMT backend for test-case generation, which includes a floating-point encoding layer that converts all floating-point operations into bit-vector operations (a process called *bit-blasting*) when encoding the program into an SMT formula. Previous ESBMC versions [8] were only able to encode and verify programs using a fixed-point representation for floating-points. This particular encoding is a valid approximation since fixed-points are used in a large number of applications in the embedded world; however, it restricted ESBMC from verifying the broad set of programs that relied on processors that implement floating-point arithmetic.

There exist various strategies to solve SMT formulae with floating-point arithmetic. It is tempting to use a real arithmetic strategy to tackle these formulae; however, the floating-point arithmetic is an approximation of the real one and introduces a new set of values (e.g., NaNs). ESBMC follows the same approach as CBMC [2] and 2LS [15], which also bit-blast all operations, including floating-point operations, before checking satisfiability using SAT solvers. The bit-blasting algorithm in ESBMC is based on the bit-blasting performed by Z3, which is an improved version of the algorithms described by Muller et al. [12]. A floating-point is encoded into SMT using a single bit-vector and follows the IEEE–754 [11] standard for the size of the exponent and significand. For instance, a half-precision floating-point (16 bits) has 1 bit for the sign, 5 bits for the exponent and 11 bits for the significand (1 hidden bit) [11]. Thus, the floating-point encoding layer in ESBMC performs the operations in the bit-vectors representing the floating-points, e.g., the formula to check if a bit-vector is a NaN checks if the exponent is all `1's` and if the significand is not zero. The resulting SMT formulae are the translation of the floating-point arithmetic digital circuits to SMT [12].

The improved SMT backend is an extension of our previous work on floating-point arithmetic encoding [9]. Previously, we extended ESBMC to encode floating-point arithmetic into SMT, however, we were restricted to SMT solvers that supported the `FP` theory natively (i.e., Z3, MathSAT and CVC4) [9]. Now, the floating-point encoding layer extends the `FP` theory support to all solvers supported by ESBMC, including Boolector [13] and Yices [4], which do not natively support that `FP` theory. In Test-Comp'20, ESBMC uses Boolector 3.0.1 and produces 470 confirmed test specifications. In particular, ESBMC achieved the the highest score in the ReachSafety-Floats, a category focused on programs with floating-point arithmetics, correctly verifying 30 out of the 32 test cases and outperforming all other tools in this category. The results in this category demonstrates the effectiveness of the floating-point bit-blasting: Boolector does not support the `FP` theory natively and yet was able to reason about almost all the test cases in the competition that involved floating-point arithmetic.

## 2 Strengths and Weaknesses

The falsification mode allows ESBMC to keep unwinding the program until a property violation is found, or until it exhausts time or memory limits. Its BMC approach, however, stops after it has found a property violation and prevents the generation of tests specifications for multiple property violations or coverage testing. This approach, however, is an advantage in the `Cover-Error` category as finding one error is the primary goal.

Encoding programs using the SMT `FP` theory has several advantages over the fixed-point approach. ESBMC can now accurately model C programs that use the IEEE floating-point arithmetic [11]. In particular, ESBMC ships with models for most of the current C11 standard functions. Furthermore, the floating-point encoding layer in ESBMC extends the support for the SMT `FP` theory to solvers that do not support it natively. ESBMC can verify programs with floating-point arithmetic using all currently supported solvers – including Boolector and Yices, which do not support the SMT `FP` theory.

In Test-Comp'20 results, 470 tests were confirmed while 13 tests were unconfirmed, where 11 were due to bugs in the script that generates the test specification (e.g., non-deterministic unions or duplication of non-deterministic values)[5], 1 was due to a bug in ESBMC that caused the tool to fail[6], and 1 was due to undefined behavior in the test case[7]. We chose Boolector for the competition because it outperforms all other SMT solvers supported by ESBMC. In the ReachSafety-Floats category, Boolector even outperforms all other SMT solvers that natively support `FP` theory. We believe that Boolector employs more abstract and less expensive techniques (e.g., algebraic reduction rules and contextual simplification) before bit-blasting SMT formulae into SAT.

The drawback of the floating-point encoding is that they are very complex; it is not uncommon to see the SMT solvers struggling to support every corner case [6,14]. The maintenance of our floating-point encoding layer is hard, and we do not yet have proof that it is entirely correct, even though empirical evidence [9] points in that direction and suggests that the approach is efficient in finding bugs as shown by Test-Comp'20 results. The complex bit-vector formulae also prevent high-level reasoning about the problem by the SMT solver, however, this is not a significant issue for ESBMC as all high-level simplifications are performed before encoding the program into SMT formulae.

## 3 Tool Setup and Configuration

In order to run our `esbmc-wrapper.py` script[8], one must set the architecture (*i.e.*, 32 or 64-bit), the competition strategy (i.e., $k$-induction, falsification, or incremental BMC), the property file path, and the benchmark path, as:

```
esbmc-wrapper.py [-a {32, 64}] [-p PROPERTY_FILE]
                 [-s {kinduction,falsi,incr,fixed}]
                 [BENCHMARK_PATH]
```

5 https://github.com/esbmc/esbmc/issues/142
6 https://github.com/esbmc/esbmc/issues/143
7 https://github.com/sosy-lab/sv-benchmarks/pull/1073
8 https://gitlab.com/sosy-lab/test-comp/archives-2020/blob/master/2020/esbmc-falsi.zip where -a sets the architecture, -p sets the property file path, and -s sets the strategy (e.g., kinduction, falsi, incr, or fixed). In Test-Comp'20, ESBMC uses falsi for falsification.

Internally, by choosing the falsification strategy, the following options are set when executing ESBMC: --no-div-by-zero-check, disables the division by zero check (required by Test-Comp); --force-malloc-success, sets that all dynamic allocations succeed (a Test-Comp requirement); --floatbv, enables floating-point SMT encoding; --falsification, enables the falsification mode; --unlimited-k-steps, removes the upper limit of iteration steps in the falsification algorithm; --witness-output, sets the witness output path; --no-bounds-check and --no-pointer-check disable bounds check and pointer safety checks, resp., since we are only interested in finding reachability bugs; --k-step 5, sets the falsification increment to 5; --no-allign-check, disables pointer alignment checks; and --no-slice, disables slicing of unnecessary instructions. The Benchexec tool info module is named esbmc.py and the benchmark definition file is esbmc-falsi.xml.

## 4 Software Project

The ESBMC source code is written in C++ and it is available for downloading at GitHub[9], which include self-contained binaries for ESBMC v6.1 64-bit. ESBMC is publicly available under the terms of the Apache License 2.0. Instructions for building ESBMC from the source code are given in the file BUILDING (including the description of all dependencies). ESBMC is an international-joint project with the SIDIA Instituto de Ciência e Tecnologia, Federal University of Amazonas, University of Southampton, University of Manchester, and the University of Stellenbosch.
